# Ciprofloxacin and Clozapine: A Potentially Fatal but Underappreciated Interaction

**DOI:** 10.1155/2016/5606098

**Published:** 2016-10-30

**Authors:** Jonathan M. Meyer, George Proctor, Michael A. Cummings, Laura J. Dardashti, Stephen M. Stahl

**Affiliations:** ^1^Department of Psychiatry, University of California, San Diego, 9500 Gilman Drive, MC 0603, La Jolla, CA 92093-0603, USA; ^2^California Department of State Hospitals (DSH), Psychopharmacology Resource Network, 3102 East Highland Avenue, Patton, CA 92369, USA; ^3^California Department of State Hospitals (DSH), Clinical Operations Advisory Council, Bateson Building, 1600 9th Street, Room 400, Sacramento, CA 95814, USA; ^4^California Department of State Hospitals (DSH), Director of Psychopharmacology Services, Bateson Building, 1600 9th Street, Room 400, Sacramento, CA 95814, USA

## Abstract

*Objective.* Clozapine provides a 50%–60% response rate in refractory schizophrenia but has a narrow therapeutic index and is susceptible to pharmacokinetic interactions, particularly with strong inhibitors or inducers of cytochrome P450 (CYP) 1A2.* Case Report.* We report the case of a 28-year-old nonsmoking female with intellectual disability who was maintained for 3 years on clozapine 100 mg orally twice daily. The patient was treated for presumptive urinary tract infection with ciprofloxacin 500 mg orally twice daily and two days later collapsed and died despite resuscitation efforts. The postmortem femoral clozapine plasma level was dramatically elevated at 2900 ng/mL, and the cause of death was listed as acute clozapine toxicity.* Conclusion.* Given the potentially fatal pharmacokinetic interaction between clozapine and ciprofloxacin, clinicians are advised to monitor baseline clozapine levels prior to adding strong CYP450 1A2 inhibitors, reduce the clozapine dose by at least two-thirds if adding a 1A2 inhibitor such as ciprofloxacin, check subsequent steady state clozapine levels, and adjust the clozapine dose to maintain levels close to those obtained at baseline.

## 1. Introduction

Clozapine remains the antipsychotic of choice for patients with treatment refractory schizophrenia and for schizophrenia patients with a history of suicidality, but it does require an attentive clinician to oversee treatment due to certain common side effects (e.g., constipation, sialorrhea, orthostasis, metabolic adverse effects, and sedation) and a small number of rare but serious issues (neutropenia, seizures, myocarditis, and cardiomyopathy) [1]. Despite the burden of adverse effects and administrative issues related to ongoing hematological monitoring, no other antipsychotic approaches clozapine's 50%–60% response rate in refractory schizophrenia using the standard definition elaborated by Kane and colleagues [1]. For typical antipsychotics, the response rate in Kane-defined refractory schizophrenia is 0%, and for olanzapine 0%–9% [2–4]. Among patients who fail olanzapine treatment, the clozapine response rate is at least 41% [5].

One contributing factor to tolerability issues is clozapine's narrow therapeutic index and its susceptibility to kinetic interactions with medications [6] or environmental exposures such as smoking [7]. To improve the likelihood of response and to avoid unnecessary toxicity, clozapine plasma level monitoring is commonly performed. General consensus recommendations indicate that the response threshold is approximately 350 ng/mL, with few patients tolerating plasma levels above 1000 ng/mL [8]. An audit of 104,127 samples collected over 7 years from 26,796 patients noted that 42.5% of specimens had levels < 350 ng/mL, 49.2% were 350–999 ng/mL, and only 8.4% were 1000 ng/mL or greater [9]. Clozapine's phase 1 metabolism occurs via multiple cytochrome P450 (CYP) enzymes including CYP 1A2, CYP 2D6, and to a lesser extent CYP 2C9 and CYP 3A4. As CYP 1A2 remains the primary pathway in most patients, a significant body of literature has accrued on the interaction with strong 1A2 inhibitor fluvoxamine starting in 1998 [10, 11]. It should be noted that fluvoxamine is also a strong inhibitor of 2C19 and a weak inhibitor of 2C8, 2C9, and 3A4, and this combination of activities is reported to increase clozapine levels 3- to 10-fold [12, 13]. Although ciprofloxacin is also a strong 1A2 inhibitor and has been in routine use for over 25 years [14], there is a paucity of information about the potential seriousness of its interaction with clozapine. Presented here is a case report in which use of ciprofloxacin in a patient on stable clozapine doses was associated with a fatal outcome.

## 2. Case Report

Ms. X was an obese 28-year-old community dwelling nonsmoking white female (weight 81.8 kg, BMI 39 kg/m^2^) with severe intellectual disability and a history of behavioral disturbances consistent with schizophrenia. After failed prior trials of other antipsychotics, she had been maintained for 3 years (2009–2012) on the combination of clozapine dissolving tablets 100 mg BID, bupropion XL 150 mg qam, escitalopram 10 mg qam, N-acetylcysteine 1200 mg BID, and memantine 10 mg BID. She was also treated with L-thyroxine for hypothyroidism, famotidine for gastroesophageal reflux, fish oil and aspirin for dyslipidemia, and fluticasone and levocetirizine for seasonal allergies. Three days before her death, she was seen in the emergency room after experiencing two episodes within the same day of feeling faint without obvious precipitants. These symptoms were noted by the house staff who initially thought the patient may have been hypoglycemic due to missing breakfast, but who subsequently brought the patient to the hospital after the 2nd event. As the patient had limited verbal abilities, the staff reported that there had been no changes in her administered medications or other habits. The vital signs showed the patient to be afebrile and normotensive, with heart rate 124/min, respiratory rate 18/min, and normal oxygen saturation. The physical examination was remarkable only for tachycardia and tachypnea without evidence of respiratory distress or use of accessory muscles. The patient was not cooperative with laboratory or radiographic evaluation and was discharged home with a recommendation of lorazepam 1 mg orally every 6 hours as needed for anxiety.

The following noon the patient appeared to be at baseline and cooperated with the trip to an outpatient laboratory which found the following abnormalities: elevated total white blood cell count of 14.7 with 84.1% neutrophils, nonfasting glucose 179 mg/dL, creatinine of 1.5 mg/dL, and CO_2_ of 16 mmol/L. Other electrolytes, serum calcium, liver function tests, and serum osmolality were normal. Urinalysis revealed trace bacteria with 3-4 WBC per high power field. A presumptive diagnosis of urinary tract infection was made, and the patient's primary care provider started her on ciprofloxacin 500 mg BID that evening. Two days later the patient collapsed at approximately 10:00 pm. Emergency services were called, cardiopulmonary resuscitation started immediately, and resuscitation efforts continued during the ambulance trip and in the emergency room until the patient was pronounced dead at 11:00 pm. A postmortem examination conducted the following morning at 9:14 a.m. found no evidence of major organ pathology with normal weight and appearance for heart, liver, kidneys, and lungs. A clozapine level obtained femorally was 2900 ng/mL (reported as 2.9 mcg/mL) and that obtained hepatically was 24,300 ng/mL (reported as 24.3 mcg/mL). The cause of death was listed as acute drug (clozapine) toxicity.

## 3. Discussion

Ciprofloxacin is a fluoroquinolone antibiotic whose broad spectrum of activity was noted in the early 1980s [22]. By 1987, kinetic interactions between ciprofloxacin and other medications, particularly theophylline, were well documented and presumed to be based on inhibition of metabolism by the responsible cytochrome P450 enzyme [23], later characterized in 1992 as CYP 1A2 [14]. For most of the 1990s, the primary CYP pathways for clozapine's phase 1 metabolism were unclear, and the CLOZARIL® package insert (PI) for 1997 states that systematic kinetic interaction studies were lacking (Table 1). Language in the 1997 PI focuses on protein binding, CYP 2D6 polymorphisms and interactions; no other isoenzymes are named. The August 2001 PI reflects advancing knowledge in this area and not only mentions CYP 1A2, 2D6, and 3A4 but also discusses a 14-day interaction study with fluvoxamine in which plasma concentrations of clozapine + metabolites increased 3-fold. During this same time frame, the first reports of interactions between clozapine and ciprofloxacin were published by Markowitz et al. in 1997 [15], Raaska and Neuvonen in 2000 [16], and Gex-Fabry et al. in 2001 [17] (Table 3). While the first two publications involved low doses of either clozapine or ciprofloxacin, the case description by Gex-Fabry et al. revealed the potential for a serious kinetic interaction, with clozapine levels increasing by a factor of 3.4 to 1218 ng/mL and a significant shift in the clozapine to N-desmethylclozapine (DMC) ratio from 1.82 to 3.33 [17]. This potential interaction with ciprofloxacin was formally acknowledged by the US Food and Drug Administration (FDA) in 2005 and is reflected in a change in PI wording for CLOZARIL in December 2005 (Table 1). This interaction was also acknowledged more broadly in the medical literature, with reviews in 2006 and 2007 commenting on the fact that ciprofloxacin ranked among the most potent CYP 1A2 inhibitors, thus placing clozapine treated patients at risk for adverse effects when combined with ciprofloxacin [6, 24]. Given the limited number of publications involving comedication with ciprofloxacin, none of these reviews raised the risk of fatality nor offered any new data beyond that in the 3 earlier case reports. Three subsequent case reports by Sambhi et al. in 2007 [18], Brownlowe and Sola in 2008 [19], and Brouwers et al. in 2009 [20] documented that a kinetic interaction between these two medications could lead to levels that potentially might be fatal, although no fatalities occurred. Starting in July 2013, the United States entered the era of structured PIs with FDA mandated language regarding the strength of inhibitors and inducers (Table 4). The revised clozapine PI (Table 1) now contained specific language on dosage modifications when adding or removing strong 1A2 inhibitors, thus incorporating knowledge about fluvoxamine and ciprofloxacin that had been in the literature for over 10 years.

While the peripheral and central postmortem clozapine levels in this case were extraordinarily high and consistent with a fatal outcome, there are two factors that must be considered: (a) this patient had a complex clinical picture and was receiving many medications; (b) postmortem redistribution effects must be accounted for to assess the true nature of ciprofloxacin's effect. Although methods for measuring clozapine and its metabolite N-desmethylclozapine (DMC) in whole blood and tissue were available by 1993 [25], the extent of postmortem redistribution of newer lipophilic antipsychotics was not recognized until 1998 with the publication of an olanzapine case report showing significantly higher cardiac and biliary levels than those from the gastric specimen [26]. The first report discussing postmortem redistribution of clozapine occurred in 2003, and the authors cast doubt on the validity of the intracardiac clozapine blood level 4500 ng/mL in a patient on a stable clozapine dose of 350 mg/d who had refused her clozapine for 24 hours before death [27]. Flanagan et al.'s paper later that year using porcine data [28] and subsequent publications from human cases outlined considerations in interpreting postmortem levels in clozapine-related deaths [29, 30] (see the following list of Flanagan's criteria). While central postmortem clozapine levels are unreliable and differ by 10-fold or more from peripheral values, even under ideal circumstances, samples obtained from femoral veins may rise as much as 1.5-fold after death [31].

*Flanagan's List of Issue to Address When Investigating Clozapine-Related Death [30]*

1. Is there circumstantial or pathological evidence of self-poisoning?
2. Is there evidence of prior recent exposure to clozapine (i.e., is the patient likely to have been tolerant of the hypotensive effects of the drug?)
3. Was blood collected postmortem by venipuncture from a peripheral vein before opening the body?
4. Was the patient prescribed any other drugs and were other drugs looked for on toxicological analysis?
5. What was the clozapine dose and dosage regimen?
6. Were tablets or suspension dispensed?
7. Did smoking habit or clozapine dosage change recently?
8. Was there a history of substance abuse?
9. Was the blood norclozapine level measured?
10. Are antemortem plasma or whole blood clozapine/norclozapine results available?
11. Was histology performed, especially heart and liver?
12. Was there evidence of pneumonia?
13. Was there clinical or postmortem evidence of vomiting, aspiration of vomit, or other GI tract problem?

As the death in this case occurred in hospital and the autopsy was performed within 10 hours, the extent of postmortem redistribution is greatly minimized using the criteria set forth by Flanagan (see the prior list of Flanagan's criteria). Nonetheless, the fact that hepatic clozapine levels were 8-fold higher than femoral levels reflects how quickly this process can occur. Using the worst case estimate that the femoral levels may have increased up to 50% since the time of death [31], we arrive at an antemortem plasma clozapine level of 1933 ng/mL at the minimum. Since this patient did not control her own medication supply, had no history of medication hoarding, and was adherent with medications, the high plasma level is certainly the product of the interaction between clozapine and the strong CYP1A2 inhibitor ciprofloxacin started two days previously.

The one unknown is to what extent ciprofloxacin increased this patient's plasma clozapine levels. There is no record of outpatient clozapine levels obtained in the year prior to this incident, so one must infer from the clinical information an estimated level based on the dose, age, gender, weight, and status as a nonsmoker, with the added interaction from the CYP 2D6 inhibitor bupropion. The clozapine plasma level equation derived by Rostami-Hodjegan and colleagues from 4963 specimens permit calculation of an estimated plasma clozapine level with only one assumption: that this nonsmoking patient is a CYP 1A2 extensive metabolizer [32]. Using their equation, the estimated clozapine level without the bupropion interaction is 262 ng/mL. While CYP 2D6 is a minor phase 1 metabolic pathway for clozapine, strong 2D6 inhibitors increase plasma clozapine levels 40%–70% [13], with one case report of paroxetine increasing clozapine levels 100% in a patient whose baseline clozapine level was in the range of 666 ng/mL–684 ng/mL [33]. When that patient's clozapine dose was halved, paroxetine increased clozapine levels approximately 30% [33]. As this patient's estimated plasma clozapine level is <300 ng/mL, a 100% increase from bupropion is unlikely, but if we use the high value of 70% obtained from case reports with strong 2D6 inhibitors, the estimated baseline clozapine level for this patient prior to ciprofloxacin exposure was 445 ng/mL. The exposure to ciprofloxacin is thus estimated to have increased this patient's clozapine levels 4.5-fold at the minimum, resulting in the patient's demise; moreover, the extent of this interaction is consistent with that reported for fluvoxamine. Unfortunately, the absence of antemortem levels necessitates speculation, but the prior use of bupropion combined with the later exposure to ciprofloxacin both emerge as important factors to consider in this outcome.

## 4. Conclusions

Although the possibility of an interaction between clozapine and ciprofloxacin has been documented in the literature since 1997 and language in the clozapine PI has existed since 2005, the potential for a fatal outcome has not been previously reported. Clinicians prescribing medications with narrow therapeutic indices must be mindful of potentially important kinetic interactions and have a reliable resource to identify kinetic data when adding agents to high-risk medications. Should newer medications be developed that are strong CYP 1A2 inhibitors, the message from this case should be heeded: (a) baseline plasma clozapine levels must be obtained prior to adding the strong CYP 1A2 inhibitor; (b) the clozapine dose reduced by at least two-thirds (and further if the patient complains of adverse effects); (c) clozapine plasma levels must be rechecked after steady state is reached with the new medication and the clozapine dose adjusted accordingly. In patients with cognitive dysfunction or more severe intellectual disabilities, avoidance of this interaction completely appears prudent, as the patient may not be able to express their physical discomfort, potentially resulting in a serious or fatal outcome.

## Figures and Tables

**Table 1:** Sequential changes in CLOZARIL package insert warnings about pharmacokinetic interactions with ciprofloxacin.

Date	Text
June 1997	*Section: Drug Interactions* *Pharmacokinetic-Related Interactions* *General*: “The risks of using of CLOZARIL® (clozapine) in combination with other drugs have not been systematically evaluated.” “Because CLOZARIL® (clozapine) is highly bound to serum protein, the administration of CLOZARIL® (clozapine) to a patient taking another drug which is highly bound to protein (e.g., warfarin, digoxin) may cause an increase in plasma concentrations of these drugs, potentially resulting in adverse effects. Conversely, adverse effects may result from displacement of protein-bound CLOZARIL® (clozapine) by other highly bound drugs.” *Specific*: “Cimetidine and erythromycin may both increase plasma levels of CLOZARIL® (clozapine) potentially resulting in adverse effects.” “Concomitant use of clozapine with other drug metabolized by cytochrome P450 2D6 may require lower doses than usually prescribed for either clozapine or the other drug.”

August 2001	*Section: Drug Interactions* *Pharmacokinetic-Related Interactions* *General*: “Clozapine is a substrate for many CYP450 isozymes, in particular 1A2, 2D6, and 3A4. The risk of metabolic interactions caused by an effect on an individual isoform is therefore minimized. Nevertheless, caution should be used in patients receiving concomitant treatment with other drugs that are either inhibitors or inducers of these enzymes.” *Specific*: “Concomitant administration of drugs known to inhibit the activity of cytochrome P450 isozymes may increase the plasma levels of clozapine. Cimetidine, caffeine and erythromycin may increase plasma levels of CLOZARIL® (clozapine), potentially resulting in adverse effects.”

January 2002–June 2005	*No change*

December 2005	*FDA Letter (12/05/2005)*: “This ‘Changes Being Effected' supplemental new drug application provides for changes in the product labeling under PRECAUTIONS, Drug Interactions, Pharmacokinetic-Related Interactions to add ciprofloxacin to the list of drugs that may increase plasma levels of CLOZARIL, potentially resulting in adverse effects.” *Section: Drug Interactions* *Pharmacokinetic-Related Interactions* *Specific*: “Concomitant administration of drugs known to inhibit the activity of cytochrome P450 isozymes may increase the plasma levels of clozapine. Cimetidine, caffeine, citalopram, ciprofloxacin, and erythromycin may increase plasma levels of CLOZARIL, potentially resulting in adverse effects.”

June 2008–March 2013	*No change*

July 2013	*Dosage and Administration: Section 2.6* Dosage Adjustments with Concomitant use of CYP1A2, CYP2D6, CYP3A4 Inhibitors or CYP1A2, CYP3A4 Inducers “Dose adjustments may be necessary in patients with concomitant use of: strong CYP1A2 inhibitors (e.g., fluvoxamine, ciprofloxacin, or enoxacin); moderate or weak CYP1A2 inhibitors (e.g., oral contraceptives, or caffeine); CYP2D6 or CYP3A4 inhibitors (e.g., cimetidine, escitalopram, erythromycin, paroxetine, bupropion, fluoxetine, quinidine, duloxetine, terbinafine, or sertraline); CYP3A4 inducers (e.g., phenytoin, carbamazepine, St. John's wort, and rifampin); or CYP1A2 inducers (e.g., tobacco smoking) (Table 2).” *Drug Interactions: Section 7.1* Potential for Other Drugs to Affect CLOZARIL *CYP1A2 Inhibitors*: “Concomitant use of CLOZARIL and CYP1A2 inhibitors can increase plasma levels of clozapine, potentially resulting in adverse reactions. Reduce the CLOZARIL dose to one third of the original dose when CLOZARIL is coadministered with strong CYP1A2 inhibitors (e.g., fluvoxamine, ciprofloxacin, or enoxacin). The CLOZARIL dose should be increased to the original dose when coadministration of strong CYP1A2 inhibitors is discontinued *[see Dosage and Administration (2.6), Clinical Pharmacology (12.3)]*.”

*Source*: http://www.accessdata.fda.gov/scripts/cder/drugsatfda/index.cfm?fuseaction=Search.Label_ ApprovalHistory#apphist *accessed 07/01/2016*.

**Table 2:** US July 2013 CLOZARIL package insert table on dose adjustment in patients taking concomitant strong CYP 1A2 inhibitors.

Comedications	Scenarios		
	Initiating CLOZARIL while taking a comedication	Adding a comedication while taking CLOZARIL	Discontinuing a comedication while continuing CLOZARIL
Strong CYP1A2 inhibitors	Use one third of the CLOZARIL dose		Increase CLOZARIL dose based on clinical response

**Table 3:** Published reports of ciprofloxacin-clozapine interaction.

Author, year	Description
Markowitz et al.1997 [15]	Case report: 72-year-old male with multi-infarct dementia and diabetes mellitus, on low dose clozapine for behavioral management (18.75 mg/d). Ciprofloxacin 500 mg BID started for leg ulcer. Ten days later the patient developed agitation, requiring hospitalization. At time of admission the clozapine level was 90 ng/mL. After ciprofloxacin was discontinued, clozapine levels dropped below the limit of detection (50 ng/mL).
Raaska and Neuvonen 2000 [16]	Randomized, double-blind, cross-over study in 7 schizophrenia inpatients who volunteered to receive either 250 mg BID ciprofloxacin or placebo BID for 7 days. Ciprofloxacin increased mean serum concentration of clozapine and DMC by 29% (*P* < 0.01) and 31% (*P* < 0.05), respectively. There was a significant positive correlation (*r* = 0.90, *P* < 0.01) between the ciprofloxacin level and increase in total concentration of clozapine + DMC.
Gex-Fabry et al. 2001 [17]	Case report: 46-year-old male with schizophrenia on clozapine 400 mg/d with adherence issues, but whose highest plasma levels in the prior 6 months for clozapine and DMC were 500 ng/mL and 375 ng/mL, respectively. Levels obtained on a clozapine dose of 500 mg/d before ciprofloxacin was introduced were clozapine 354 ng/mL; DMC 194 ng/mL. The ratio of clozapine to DMC was 1.82. The clozapine dose was increased to 775 mg/d (a 1.6-fold change), and later ciprofloxacin 1500 mg/d was started for a UTI. The clozapine and DMC levels increased to 1218 ng/mL and 371 ng/mL, respectively, a 3.4-fold change for clozapine. The clozapine/DMC ratio increased to 3.33. Repeat clozapine and DMC levels after 3 days on ciprofloxacin 3000 mg/day and clozapine 775 mg/day were 1197 ng/mL and 475 ng/mL, respectively. Nine days after discontinuing ciprofloxacin, clozapine and DMC levels decreased 39% and 54%, respectively, to 730 ng/mL and 256 ng/mL after only a 19% drop in the clozapine dose to 600 mg/day.
Sambhi et al. 2007 [18]	Case report: 47-year-old male, chronic inpatient with schizophrenia, managed on clozapine 750 mg/d along with amisulpride 450 mg/d and lorazepam 4.5 mg/d. Patient developed acute epididymo-orchitis for which ciprofloxacin was recommended, but at the lower dose of 500 mg BID instead of 750 mg BID to minimize kinetic interactions with clozapine. The serum clozapine level on the day before ciprofloxacin was started was 550 ng/mL but rose to 2570 ng/mL after 4 days of treatment with ciprofloxacin. The clozapine dose was reduced 40% to 450 mg/d, and the repeat level on day 14 of ciprofloxacin treatment was 130 ng/mL. There was no change in smoking status.
Brownlowe and Sola 2008 [19]	Case report: 64-year-old female with schizophrenia on long-term clozapine therapy was admitted to a hospital for treatment of urosepsis. When the admission EKG showed prolonged QTc and an echocardiogram revealed hypokinesia, clozapine was stopped. When clozapine was resumed one month later after other antipsychotics failed, the patient exhibited no issues with intolerance. However, 4 months later she was readmitted with mental status changes after starting on ciprofloxacin for another UTI. The clozapine level was elevated at 1498 ng/mL.
Brouwers et al. 2009 [20]	Case reports: Case One: 46-year-old male with schizophrenia on clozapine 900 mg/d was admitted to a hospital for treatment of urosepsis. He received intravenous ciprofloxacin 400 mg BID for 4 days and was discharged without adverse effects, but the patient did lose 7 kg during the stay. Three days later, he was admitted with rhabdomyolysis. Labs revealed a CK of 195,000 U/L and abnormal liver function tests. The clozapine level obtained 24 hours after stopping clozapine was 890 ng/mL, but no levels were obtained during ciprofloxacin treatment. The authors deemed the interaction with ciprofloxacin probable. Case Two: 58-year-old male with schizophrenia on clozapine 300 mg/d was admitted to a hospital for treatment of urosepsis. He received 2 days of intravenous ciprofloxacin 200 mg BID for 4 days before ciprofloxacin was stopped due to concerns about the interaction with clozapine. Clozapine plasma levels measured prior to and 3 days after the start of ciprofloxacin were 850 ng/mL and 1720 ng/mL, respectively. The authors deemed the interaction with ciprofloxacin probable.

DMC: N-desmethylclozapine; CK: creatine kinase; UTI: urinary tract infection.

**Table 4:** US FDA classification of in vivo cytochrome P450 inhibitors by impact on area under the curve (AUC) and clearance [21].

Strength of interaction	Definition
Strong	A strong inhibitor for a specific CYP is defined as an inhibitor that increases the AUC of a substrate for that CYP by equal to or more than 5-fold (or >80% decrease in clearance)
Moderate	A moderate inhibitor for a specific CYP is defined as an inhibitor that increases the AUC of a sensitive substrate for that CYP by less than 5-fold but equal to or more than 2-fold (or 50%–80% decrease in clearance)
Weak	A weak inhibitor for a specific CYP is defined as an inhibitor that increases the AUC of a sensitive substrate for that CYP by less than 2-fold but equal to or more than 1.25-fold (or 20%–50% decrease in clearance)
